# A case report of abscopal toxicity in a patient with lung adenocarcinoma

**DOI:** 10.1093/omcr/omad076

**Published:** 2023-07-18

**Authors:** Aswanth Reddy, Nkolika Nwankwo, Arjun Sekar, Aswini Kumar

**Affiliations:** Department of Hematology and Oncology, Mercy Clinic, 7001 Rogers ave, Fort Smith, AR 72903, USA; Department of Internal Medicine, Mercy Clinic, 7001 Rogers ave, Fort Smith, AR 72903, USA; Department of Nephrology, Rochester Regional Health, RGH Center for Kidney Disease and Hypertension, 370 Ridge Rd E, Ste 20, Rochester, NY 14621, USA; Department of Cardiology, Mercy Clinic, 7001 Rogers ave, Fort Smith, AR 72903, USA

## Abstract

The abscopal effect describes tumor responses outside the irradiated field. The literature shows increased overall survival and response rates in patients receiving immunotherapy and radiation, likely from exaggerated abscopal effects. We present a 57-year-old woman with stage 4 lung adenocarcinoma who received treatment with a combination of chemotherapy and immunotherapy. She had disease progression on maintenance immunotherapy, confirming resistance. Palliative radiation to the sternal bone lesion resulted in a significant response to all areas of cancer, confirming the abscopal effect. Unfortunately, she developed severe pneumonitis; to our knowledge, this is the first case of abscopal lung toxicity.

## INTRODUCTION

The word abscopal was first used by Mole in 1953, meaning ‘at a distance from the irradiated volume but within the same organism’ [1]. The abscopal effect describes tumor responses outside the irradiated field, a rare phenomenon. A recent literature review of the abscopal effect between 1969 and 2014 detected only 46 cases, even though millions of patients are being treated worldwide with radiation [2]. Local radiation plays a wide range of roles in cancer treatment and helps reduce the risk of recurrence after surgery, curative treatment and palliation of symptoms. Ionizing radiation (IR) causes DNA damage by several mechanisms, such as producing reactive oxygen species and DNA double-stranded breaks. The latter is the principal mechanism by which IR induces DNA damage. There is evidence of increased overall survival and response rates in patients receiving immunotherapy and radiation, likely from exaggerated abscopal effects, and several prospective studies are trying to study this phenomenon.

## CASE REPORT

**Figure 1:**
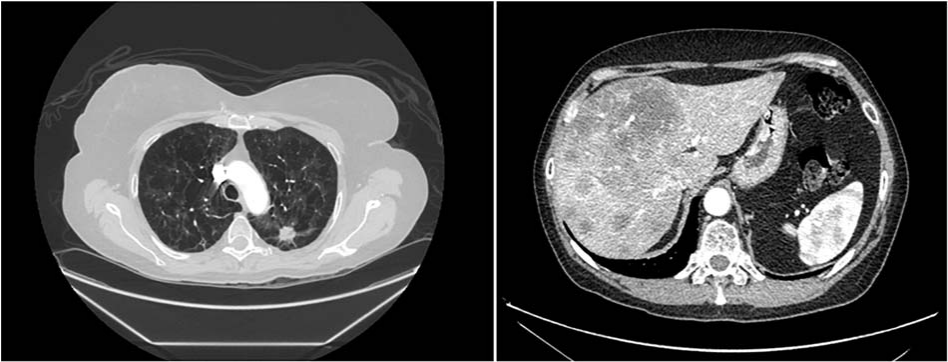
Computerized tomography showing left lung spiculated nodule and multiple liver metastatic lesions.

A 57-year-old female with a 37-pack-year smoking history presented to the emergency department complaining of right-sided abdominal pain. Ultrasound showed a heterogeneous liver with numerous nodules concerning metastatic disease. Computed tomography (CT) of the chest, abdomen and pelvis showed a 1.8 cm spiculated left upper lobe lung nodule, several lytic bone lesions and extensive hepatic metastatic disease (Fig. 1).

**Figure 2:**
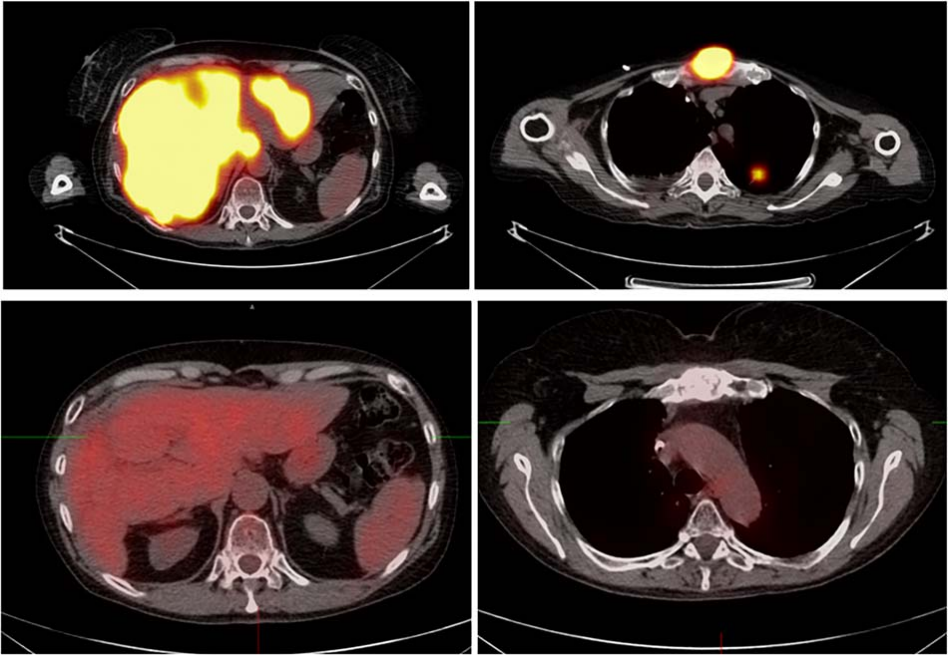
Positron emission tomography **(A)** Showing diffuse liver metastasis, lung nodule and a sternal bone metastatic lesion confirming disease progression. **(B)** Eight weeks of radiation therapy showing resolution of activity in the liver, lung nodule and sternal bone lesion.

**Figure 3:**
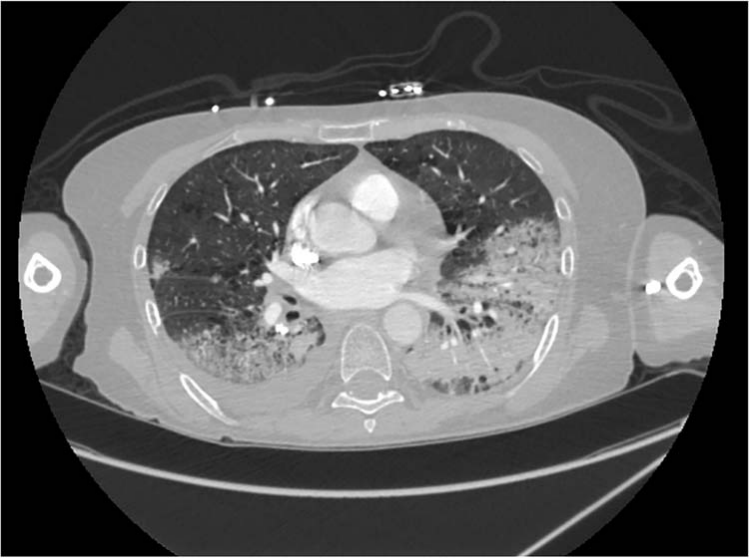
Computerized tomography showing areas of consolidation bilaterally consistent with pneumonitis.

A biopsy of the liver lesion showed nests and clusters of polygonal-shaped tumor cells with irregular nuclei and copious amounts of finely vacuolated cytoplasm. Immunoperoxidase stains revealed positive cytokeratin and thyroid transcription factor-1. Tumor cells were negative for p40 and hep par-1 stains, consistent with metastatic adenocarcinoma from lung primary. Laboratory data showed elevated liver enzymes and carcinoembryonic antigen (CEA). Next-generation sequencing of the tumor revealed no actionable mutation, PD-L1 ˂1% with a tumor mutational burden (TMB) of 97th percentile. She started carboplatin, pemetrexed and pembrolizumab with monthly denosumab injections. After four treatment cycles restaging scan showed a treatment response, and she continued maintenance therapy with pemetrexed and pembrolizumab. Four months on maintenance therapy, the CEA started trending up, and she noticed worsening fatigue and sternal pain. Pemetrexed was discontinued to help with fatigue. A PET-CT scan showed significant progression with left upper lobe primary carcinoma, an extensive hepatic metastatic disease with most liver parenchyma replaced by tumor and progressive bone metastasis, including a large lesion in her sternal bone (Fig. 2). She started palliative radiation for the sternal lesion and received 2500 cGy in five fractions while continuing pembrolizumab. Clinically she noticed significant resolution of sternal pain and fatigue and decided against changing systemic therapy. A total of 8 weeks after radiation, a bone scan revealed reduced activity in the sternum. However, a PET-CT scan surprisingly showed significant improvement in liver lesions (Fig. 2) and a corresponding improvement in CEA. One month later, she was admitted to the hospital for dyspnea and severe hypoxia, with CT showing evidence of pneumonitis (Fig. 3). Grade 2 immunotherapy-induced pneumonitis was suspected, and immunotherapy was held. She received a tapering systemic corticosteroid regimen, steadily improving her symptoms. Immunotherapy was rechallenged per guideline-directed management (American Society of clinical oncology—Management of Immunotherapy Toxicity 2021). Unfortunately, rechallenging resulted in recurrent pneumonitis; hence, we discontinued treatment permanently. At her last follow-up, she appeared clinically well 6 months after stopping immunotherapy. A PET-CT showed no fluorodeoxyglucose activity in the liver, lungs and bones.

## DISCUSSION

The natural anticancer response is partly mediated by dendritic cells presenting new antigens to T cells [3]. Cancer cells evade the body’s immune response by impairing T-cell activity and dendritic-cell mediated stimulation of programmed cell death ligand (PD-L1). T-cell immune checkpoint inhibitors that overcome this include anti-programmed cell death protein 1, anti-PD-L1 and anti-cytotoxic T-lymphocyte–associated antigen 4 monoclonal antibodies [4], commonly termed immunotherapy. Immunotherapy has been particularly promising in patients with high PD-L1 scores and higher tumor mutational burden [5]. Our patient had a high TMB, and she had an expected initial response to pembrolizumab. One of the crucial factors for the abscopal effect is the presence of infiltrating T cells; the higher presence has a positive association with the impact. Radiation therapy is well known to induce DNA damage which is believed to be responsible for triggering the immune system but is also a central mechanism for initiating the abscopal effect [6]. The damaged cells release neo-antigens that prime and activate the cluster of differentiation 8+ T cells, which migrate and infiltrate the metastases outside the irradiated field, resulting in tumor regression.

A study of lung cancer patients treated with pembrolizumab showed improved survival when they had a history of previous radiation compared to patients who did not receive radiation [7]. The PACIFIC trial evaluating consolidation durvalumab after chemoradiation for unresectable stage III non-small cell lung cancer (NSCLC) patients showed better survival outcomes in patients receiving immunotherapy without significantly increasing immune-related side effects [8]. In contrast, a study of patients with NSCLC treated with concurrent immunotherapy (durvalumab–tremelimumab) and radiation showed no significant difference in patients receiving different doses of radiation [9]. Note that the patients in this study were pretreated with immunotherapy, and these findings raise doubts about radiation as a modality to overcome immunotherapy resistance. Despite contrary observations, our patient had an excellent abscopal response after palliative radiation and likely overcame immunotherapy resistance. Similar observations have been noted in multiple case studies. A unique feature of our case is diffuse pneumonitis that we firmly believe is an abscopal toxicity from systemic immune activation after radiation outside the lung. We described this unique phenomenon of distant effect as ‘abscopal toxicity’ (toxicity in the organ not exposed to irradiation field) since this finding is novel and has not been described in the literature. Our patient tolerated immunotherapy well without any side effects until she received radiation. Another critical factor is that our patient received palliative dose radiation to the sternum and never received radiation to the lung fields. We did not identify any case of abscopal pneumonitis in our literature search.

Regarding other abscopal toxicities, we found one other case reported in a patient exhibiting bullous pemphigoid after receiving palliative radiation while on treatment with nivolumab for renal cell cancer [10]. We believe it is essential to be aware of abscopal toxicity as some of these toxicities can appear at a distant site and result in significant morbidity. Immunotherapy resistance is an area that needs timely research as many patients stop responding after some time. Although case studies like ours suggest radiation could overcome the resistance, there is a need for an accurate biomarker to identify patients who will benefit from this.

## Data Availability

Data is available on request to the corresponding author.
